# How sports-implied packaging of protein powder products enhances the purchase intention of Generation Z: evidence from multiple experiments

**DOI:** 10.3389/fnut.2025.1645614

**Published:** 2025-11-14

**Authors:** Chang Hu, Junhao Bin, Wen Zhang, Wenying Huang

**Affiliations:** 1 School of Physical Education, Jiangxi Normal University, Nanchang, China; 2 Food College of Shaoguan University, Shaoguan, China

**Keywords:** protein powder products, sports-implied packaging, Generation Z, purchase intention, exercise motivation, psychological empowerment, disease threat

## Abstract

**Background:**

In recent years, Generation Z's demand for protein powder and other sports nutrition products has grown significantly. While existing studies have primarily focused on how product functionalities influence consumer decisions, the decision-triggering mechanisms of packaging design within the framework of sensory marketing theory remain unclear. Specifically, the psychological pathways through which sports-implied elements influence Generation Z's purchasing decisions remain underexplored.

**Method:**

Based on embodied cognition theory and self-determination theory, this study employs a mixed research methodology to conduct three online scenario experiments (*N* = 1,456). We examined the impact of sports-implied packaging on Generation Z's purchase intention using two-way ANOVA and Process Model 6 to test the moderating role of disease threat and the chained mediating roles of exercise motivation and psychological empowerment.

**Results:**

Compared to traditional packaging, sports-implied packaging significantly enhances Generation Z's purchase intention. In this process, Generation Z's exercise motivation and psychological empowerment further amplify the stimulating effect of sports-implied labels, strengthening their purchase intention. Notably, the intensifying role of intrinsic motivation is particularly pronounced. Additionally, higher levels of disease threat magnify the promoting effect of sports implications.

**Conclusion:**

This study provides theoretical support for protein powder manufacturers to develop precise marketing strategies. It is recommended that companies optimize packaging designs based on disease threat levels, psychological empowerment, and exercise motivations based on their target demographics.

## 1 Introduction

In consumer environments, product packaging functions as more than a channel for conveying information (1, 2); it serves as a powerful perceptual cue that automatically guides cognition and behavior (3). Sports implications exemplify such cues, as visual and tactile elements can implicitly activate sports-related associations and influence behavioral intentions. Grounded in embodied cognition, this phenomenon emphasizes the close interconnection between perception and bodily experience, whereby visual stimuli can prime processes related to action (4). From this perspective, packaging is not a passive container but an embodied stimulus capable of shaping consumers' psychological and behavioral responses (5). Extant research demonstrates that exposure to both environmental and social sport-related cues, such as observing others' exercise behaviors or interacting with performance-oriented equipment, can increase motivation and participation (6). Nevertheless, the potential of embodied packaging cues to enhance consumer behavior remains insufficiently studied.

Protein powder provides an ideal context for exploring this effect. Unlike general supplements, protein powders are inherently associated with fitness goals and body management (7). The market has expanded rapidly in recent years, with Generation Z emerging as a key consumer segment characterized by heightened health awareness and sensitivity to visual aesthetics (8). This demographic responds strongly to sensory stimulation delivered through packaging, which suggests that the embodied design of sports-implied packaging may significantly influence their purchase intentions (9). However, existing protein powder packaging often depends on conventional informational cues, such as ingredient lists and nutritional tables (10), and is less effective at activating sports-related identities compared with more dynamic embodied stimuli.

To explain why sports-implied packaging may foster stronger purchase intentions, this study integrates embodied cognition with self-determination theory (11). Packaging cues are conceptualized as embodied stimuli: dynamic visuals, athletic imagery, and tactile textures simulate sport-related experiences and thereby fulfill psychological needs for autonomy and competence. In turn, packaging enhances psychological empowerment, strengthens exercise motivation, and ultimately drives purchase intention (12). This mediating pathway reflects the embodied nature of perception and action, in which external sensory input becomes translated into internal motivation and consumer behavior.

Despite extensive literature on labeling and packaging effects (13, 14), the embodied role of sports implications in the protein supplement domain has not been systematically investigated. Prior studies have largely emphasized informational or symbolic cues while neglecting how packaging can function as a bodily stimulus that simultaneously activates identity, empowerment, and motivation. To bridge this gap, the present study adopts embodied cognition as its primary framework to examine how sports-implied packaging shapes Generation Z consumers' purchase intentions for protein powder. By emphasizing the main effect and clarifying the mediating pathway through empowerment and motivation, this study extends theoretical understanding of embodied consumer responses and offers practical implications for designing packaging that resonates with health-conscious younger consumers.

## 2 Theoretical framework and hypotheses

### 2.1 The relationship between sports-implied packaging of protein powder products and Generation Z's purchase intention

Embodied cognition theory posits that cognitive processes are grounded in sensorimotor experiences, such that environmental stimuli automatically trigger perceptual simulations of associated concepts (15). Visual sports cues, such as images of athletes or dynamic action scenes, can therefore activate embodied simulations of energy, vitality, and performance-related states (16). Once activated, these simulations foster approach-oriented behaviors by aligning with individuals' pursuit of health and performance goals. In this sense, sports-implied packaging provides more than aesthetic decoration; it operates as an embodied prime that channels consumers' implicit associations into readiness for goal-congruent action.

In parallel, sensory marketing research underscores packaging's power as a multisensory stimulus (17). Packaging shapes over 70% of in-store purchase decisions by providing visual, tactile, and symbolic cues that reduce cognitive load and heighten hedonic appeal (18). Prior studies confirm that dynamic visual attributes such as vivid color, shape, or ergonomic design can increase perceived vitality and strengthen purchase intention (19). However, most of this work concentrates on static perceptual features (20, 21) and pays little attention to thematic and embodied cues that activate rich action-oriented simulations. This oversight is pronounced in protein powder marketing, where packaging has historically emphasized factual information (ingredients, nutritional facts, certifications) rather than embodied vitality cues.

This creates a clear research gap: while sports cues have been shown to stimulate exercise participation in physical environments (e.g., gym layouts increasing workout frequency), it is still unclear whether sports-implied packaging can translate these embodied effects into consumer behavior, specifically purchase intentions for nutrition products. Given Generation Z's elevated health awareness and sensitivity to visual aesthetics (22), sports-implied packaging has the potential to prime stronger approach behaviors than traditional, information-heavy designs.

Based on the aforementioned analysis, this study proposes the following hypothesis:

H1: Compared to traditional packaging, protein powder products with sports-implied elements are more likely to generate purchase intention among Generation Z.

### 2.2 The chain mediating roles of psychological empowerment and exercise motivation

Psychological empowerment refers to an individual's perceived sense of control, competence, and meaning in relation to their environment (23). In consumer contexts, empowerment is fostered when external cues enhance consumers' confidence in their ability to make effective choices, which in turn increases purchase intentions (24, 25). For Generation Z, who value autonomy and goal alignment, products that strengthen self-efficacy and resonate with identity are especially appealing (26, 27). Sports-implied packaging, by incorporating athletic bodies, dynamic visuals, and health-related motifs, symbolically conveys that strength and vitality are attainable. These cues help satisfy consumers' psychological needs for competence (the athletic body is within reach) and autonomy (choosing the product advances personal goals). In doing so, such packaging embodies empowerment.

Self-determination theory provides a useful framework to explain how empowerment translates into motivation. The theory distinguishes between intrinsic and extrinsic motivation (28). Intrinsic motivation arises from interest, enjoyment, and self-efficacy, whereas extrinsic motivation is driven by external rewards or pressures (29). Prior studies consistently show that intrinsic motivation sustains long-term behaviors more effectively than extrinsic drivers, particularly in health and sports consumption (30, 31). In packaging contexts, sports-related imagery can strengthen psychological empowerment and, through this lens, enhance consumers' intrinsic motivation by reinforcing self-determined reasons for engaging in health behaviors. Empowered individuals thus feel competent and autonomous, which increases their willingness to purchase health-oriented products.

Despite these insights, research has rarely considered how packaging simultaneously enhances psychological empowerment and motivation. Most studies test either empowerment or motivation separately (32, 33) without examining their chain-mediating role between sensory cues and consumer purchase intention. By integrating embodied cognition and self-determination theory, we argue that sports-implied packaging cues activate embodied simulations, which empower consumers, enhance their intrinsic or extrinsic motivation, and ultimately drive purchase intention.

Based on the aforementioned analysis, this study proposes the following hypotheses:

H2a: Psychological empowerment and intrinsic motivation mediate the relationship between sports-implied packaging of protein products and Generation Z's purchase intention.
H2b: Psychological empowerment and extrinsic motivation mediate the relationship between sports implications and Generation Z's purchase intention.
H3: Compared to extrinsic motivation, the indirect effect of psychological empowerment and intrinsic motivation is more significant.

### 2.3 The moderating role of disease threat

Disease threat refers to an individual's perception of health risks, which can substantially influence health-related behaviors and purchasing decisions (34). The Health Belief Model emphasizes that individuals' perceptions of susceptibility and severity are key drivers of preventive behaviors (35, 36). While HBM provides a useful foundation, more recent frameworks such as the Extended Parallel Process Model (EPPM) and Terror Management Theory (TMT) offer deeper insights into how perceived threats shape consumer decisions.

According to the EPPM, when individuals perceive a health threat, they evaluate both the severity of the threat and the efficacy of the response (37). If the response is perceived as effective, individuals engage in danger control behaviors—taking constructive action to mitigate the risk. In the context of sports nutrition, protein powder products with sports-implied packaging may be perceived as an efficacious response, helping consumers strengthen their bodies and protect against health threats. Conversely, if individuals perceive high threat but low response efficacy, they may engage in fear control behaviors, such as denial or avoidance. Effective packaging thus plays a critical role by signaling efficacy and channeling health threat into purchase intention.

Similarly, Terror Management Theory (TMT) posits that awareness of disease or mortality threats motivates individuals to engage in behaviors that reinforce self-esteem and cultural values (38). For Generation Z, health-consciousness and fitness are salient cultural values (39), and sports-implied packaging directly connects the product to these values. By embodying vitality, performance, and resilience, such packaging allows consumers to symbolically manage existential concerns, resulting in stronger purchase intentions under heightened disease threat.

Taken together, these perspectives suggest that disease threat functions as a boundary condition that amplifies the effects of sports-implied packaging. For Generation Z consumers, heightened perceptions of disease risk make them more receptive to embodied cues of health and vitality. In such contexts, sports-implied packaging is more likely to activate danger control responses (EPPM) and provide symbolic value for managing mortality concerns (TMT), thereby strengthening the link between packaging design and purchase intention.

**Figure 1:**
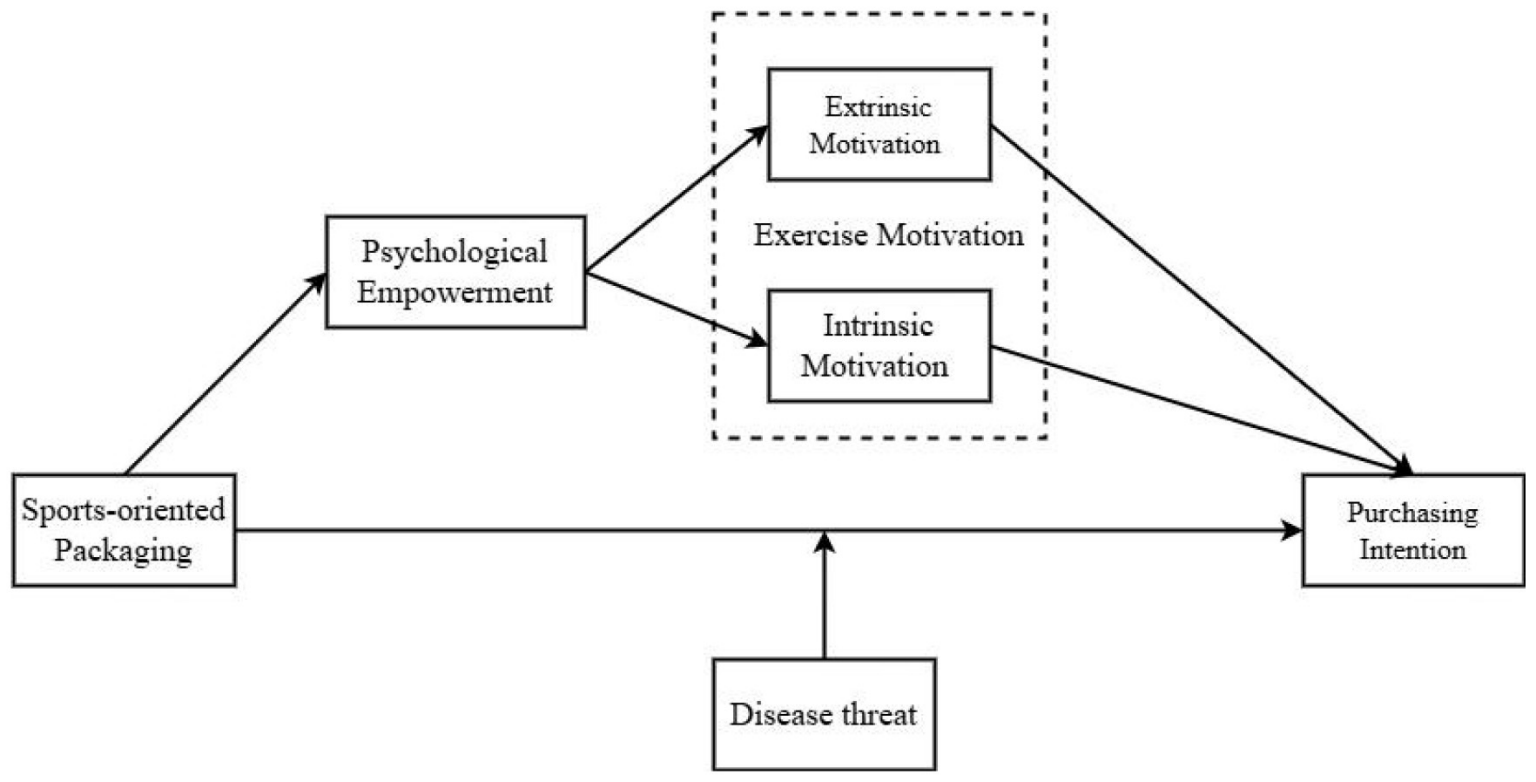
Theoretical framework of the study.

Based on the aforementioned analysis, this study proposes the following hypothesis:

H4: When Generation Z feels a higher disease threat, protein powder products with sports-implied packaging tend to have a higher purchase intention. The theoretical model of this study is illustrated in Figure 1.

## 3 Research overview

This study conducted four related experiments to further verify the above three hypotheses. Specifically, in Experiment 1, we analyzed the impact of sports-implied packaging of protein products on Generation Z's purchase intention, validating Hypothesis H1. Experiment 2 examined the chained mediating roles of psychological empowerment and exercise motivation in the relationship between sports-implied packaging and Generation Z's purchase intention, validating Hypotheses H2 and H3. In Experiment 3, we explored the moderating role of disease threat in the relationship between sports-implied packaging and Generation Z's purchase intention, validating Hypothesis H4. This study designed different packaging for two protein products to further manipulate the effectiveness of sports-implied packaging for protein products.

We informed all participants about the purpose of the study, its benefits, potential risks, and asked whether they wished to participate. After signing the informed consent form, participants completed the relevant experiments and received a remuneration of 3 CNY. The Academic Ethics Committee of Jiangxi Normal University reviewed and approved this study.

**Figure 2:**
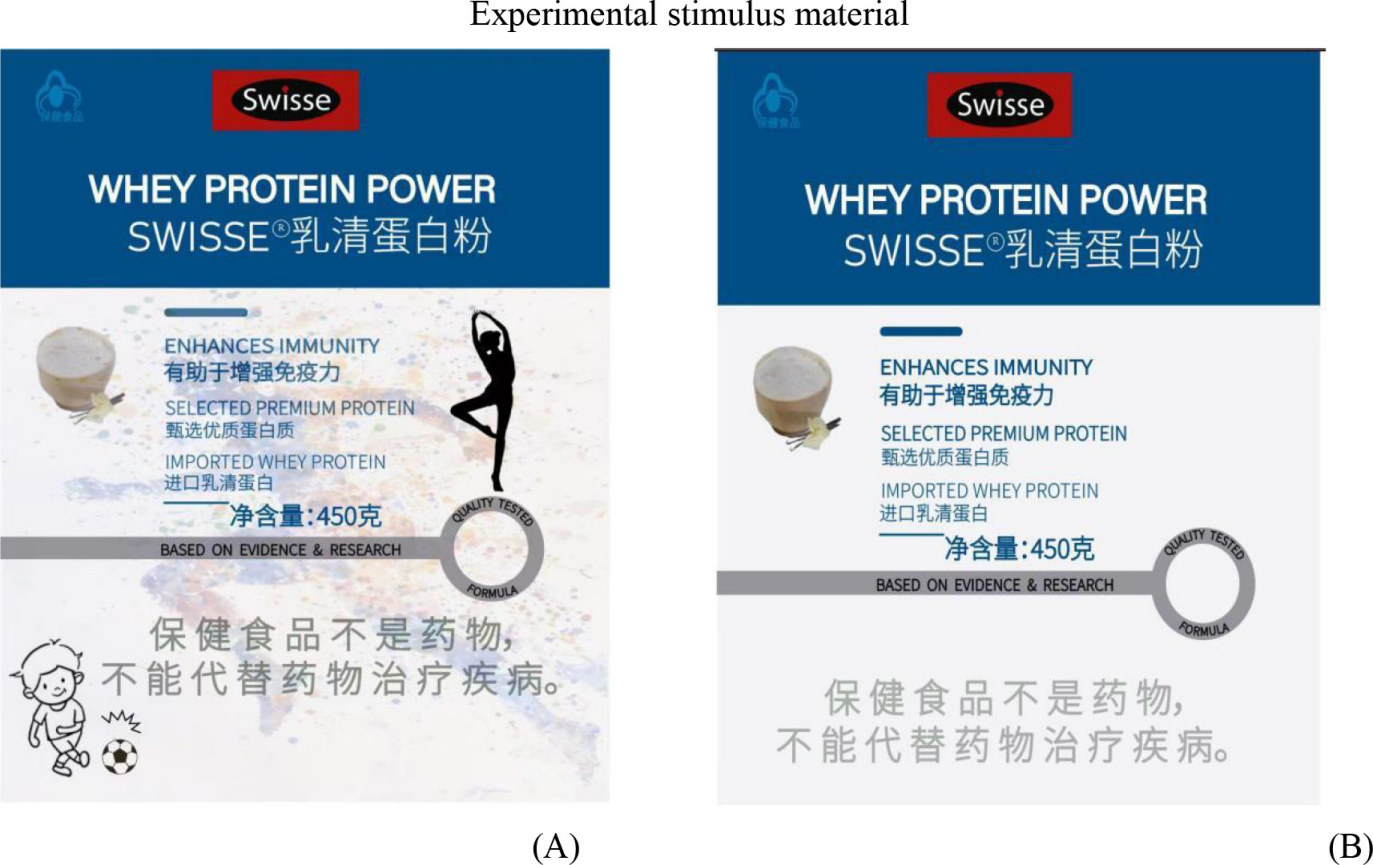
Experimental stimulus materials: **(A)** sports-implied packaging and **(B)** traditional packaging.

The experimental designs for the three studies are outlined in Table 1. The demographic information for the three studies is presented in Table 2. The experimental stimulus materials are shown in Figure 2.

**Table 1:** Research designs of the three experiments.

**Experiment**	**Experiment 1**	**Experiment 2**	**Experiment 3**
Purpose	To test for the main effect (H1)	To test the chain mediation effect of psychological empowerment and exercise motivation (H2)	To test the moderating effect of disease threat (H3)
Independent variable	Sports-implied packaging	Sports-implied packaging	Sports-implied packaging
Dependent variable	Purchase Intention	Purchase Intention	Purchase Intention
Mediators	–	Psychological empowerment and exercise motivation	–
Moderator	–	–	Disease threat
Methods	ANOVA	ANOVA	ANOVA
		PROCESS 6	Two-way ANOVA
Results	Supported H1	Supported H2	Supported H3

**Table 2:** Demographic characteristics of participants across experiments.

**Variable**	**Items**	**Experiment 1 (*****N*** = **326)**		**Experiment 2 (*****N*** = **594)**		**Experiment 3 (*****N*** = **536)**	
		**Frequency**	**Proportion**	**Frequency**	**Proportion**	**Frequency**	**Proportion**
Gender	Male	167	51.2%	290	48.8%	281	52.4%
	Female	159	48.8%	304	51.2%	255	47.6%
Education background	Primary school	5	1.5%	8	1.3%	9	1.7%
	Junior high school	26	8.0%	6	1.0%	25	4.7%
	Technical secondary school, High school	85	26.1%	176	29.6%	65	12.1%
	Junior college	84	25.8%	200	33.7%	210	39.2%
	Undergraduate college	96	29.4%	186	31.3%	206	38.4%
	Postgraduate	28	8.6%	16	2.7%	17	3.2%
	Doctor-postgraduate	2	0.6%	2	0.3%	4	0.7%
Place of residence	Urban Area	185	56.7%	349	58.8%	318	59.3%
	Suburban Area	141	43.3%	245	41.2%	218	40.7%
Exercise habit	Less than once a week	139	42.6%	252	42.4%	305	56.9%
	2 to 3 times	156	47.9%	188	31.6%	188	35.1%
	More than four times	31	9.5%	154	25.9%	43	8%
Age	M ± SD	23.604 ± 3.445		23.544 ± 3.227		23.435 ± 2.953	

Prior to the main experiments, a pilot study was conducted to verify the validity of the stimulus materials. Using convenience sampling, 40 participants from Jiangxi Normal University were randomly assigned to the experimental group (Figure 2A) or the control group (Figure 2B). They evaluated the packaging on four dimensions: sports implication, color attractiveness, design complexity, and perceived quality.

Independent-samples *t*-tests showed that only sports implication differed significantly between groups (*M*_sports-*impliedgroup*_ = 5.580, SD = 1.182; *M*_traditionalpackaginggroup_ = 3.400, SD = 1.429; *t* = 5.908, *P* < 0.001), while no significant differences emerged in color attractiveness, design complexity, or perceived quality (all *ps* > 0.05). These findings confirm that the stimuli differed solely in terms of sports implication, excluding potential interference from unrelated design factors (see Table 3).

**Table 3:** Independent-samples *t*-test results for pretest stimulus materials across dimensions.

**Dimension**	**Sports-implied (M ±SD)**	**Traditional (M ±SD)**	***t***	***P-*value**
Sports implication	5.580 ± 1.182	3.400 ± 1.429	5.908	< 0.001
Color attractiveness	4.700 ± 1.559	5.450 ± 0.999	−1.811	0.079
Design complexity	4.850 ± 2.183	4.600 ± 1.501	0.422	0.676
Perceived quality	4.500 ± 2.065	4.350 ± 1.694	0.251	0.803

## 4 Experiment 1: sports-implied packaging and Generation Z's purchase intention

### 4.1 Experimental design

Experiment 1 aimed to analyze the impact of sports-implied packaging of protein products (sports-implied packaging vs. traditional packaging) on Generation Z's purchase intention. We designed a single-factor between-subjects experiment (sports-implied vs. traditional packaging) and recruited 340 participants on a professional data collection platform. Fourteen subjects were excluded because they failed the attention test and took less than 3 min to answer. The demographic information for the participants is shown in Table 2. The 326 participants were randomly assigned to the sports-implied group (*N* = 163) or the traditional packaging group (*N* = 163).

Experimental procedure: we asked all participants to imagine a scenario where they urgently needed to buy a protein powder product due to a recent cold to enhance their physical condition. At the time, they were selecting a suitable protein powder product in a large shopping mall. We then showed the two groups different packaging designs for the same product. The traditional packaging group viewed a standard protein powder product (Figure 2B), while the sports-implied group viewed a product with sports-related elements (Figure 2A). Following this, we assessed the participants' perceived sense of dynamism and vitality in the packaging. For example, we asked: “When you see the above product, do you agree that its packaging is dynamic and upbeat?” (1 = strongly disagree, 7 = strongly agree). Subsequently, participants answered questions regarding their purchase intention, such as: “When you see the above product, do you agree that you would consider purchasing this product?” (1 = strongly disagree, 7 = strongly agree) (40). Finally, we collected demographic information from the participants.

### 4.2 Experimental results

Manipulation check: we used the perceived sense of dynamism and vitality as the check variable and conducted an independent *t*-test. The results showed that, compared to the traditional packaging group, participants in the sports-implied group perceived the product packaging as more dynamic and lively (*M*_traditionalpackaginggroup_ = 4.398, SD _traditionalpackaginggroup_ = 1.762; *M*_sports-*impliedgroup*_ = 4.914, SD _sports-*impliedgroup*_ = 1.701; *t* (1,324) = −2.686, *P* = 0.008). Therefore, the manipulation in Experiment 1 was successful.

Primary effect test: we used product packaging type (sports-implied vs. traditional packaging) as the independent variable and purchase intention as the dependent variable, conducting a one-way ANOVA. The results indicated that participants' purchase intention for protein products with sports-implied packaging was significantly higher than for those with traditional packaging (*M*_traditionalpackaginggroup_ = 3.632, SD _traditionalpackaginggroup_ = 1.392; *M*_sports-*impliedgroup*_ = 4.619, SD _sports-*impliedgroup*_ = 1.729; *F* (1,324) = 32.270, *P* < 0.001). Thus, compared to traditional packaging, protein products with sports-implied elements were more likely to generate purchase intention among Generation Z, validating Hypothesis H1.

Control variable exclusion: referencing the findings of previous studies (41), gender is an important factor influencing consumers' purchase intention. We controlled for gender by conducting a covariance analysis. The results showed that, between the two packaging types, gender had no significant impact on purchase intention (*F* (1,324) = 1.455, *P* = 0.229). Therefore, this study ruled out the influence of gender on the experimental results, further validating Hypothesis H1.

### 4.3 Discussion

In Experiment 1, we validated that incorporating sports-implied elements into protein product packaging significantly enhances Generation Z's purchase intention. Compared to traditional, informative packaging, this design more effectively resonates with consumers' health and positive lifestyle goals. It implies a connection between the product's functionality and improved physical performance, evoking consumers' ideal self-image and driving purchasing decisions emotionally. This packaging strategy effectively leverages consumers' motivation to pursue a healthy lifestyle to enhance product appeal.

Although Experiment 1 analyzed the relationship between sports-implied packaging and Generation Z's purchase intention, it did not further explore the internal mechanisms driving this relationship. Therefore, in Experiment 3, we introduced psychological empowerment and exercise motivation as mediating variables to analyze the chained mediating roles of psychological empowerment and exercise motivation in the relationship between sports-implied packaging and Generation Z's purchase intention.

## 5 Experiment 2: the chain mediating roles of psychological empowerment and exercise motivation

### 5.1 Experimental design

Experiment 2 analyzed the chained mediating roles of psychological empowerment and exercise motivation in the relationship between sports-implied packaging of protein products (sports-implied vs. traditional packaging) and Generation Z's purchase intention. We designed a single-factor between-subjects experiment (sports-implied vs. traditional packaging) and recruited 600 participants on a professional data collection platform. Six subjects were excluded because they failed the attention test and took less than 3 min to answer. The demographic information for the participants is shown in Table 2. The 594 participants were randomly assigned to the sports-implied group (*N* = 297) or the traditional packaging group (*N* = 297).

Experimental procedure: we asked all participants to imagine a scenario where they regularly purchased protein products as part of their daily fitness routine. They were selecting a suitable protein product in a pharmacy at the time. We then showed the two groups different packaging designs for the same product. The traditional packaging group viewed a standard protein powder product (Figure 2B), while the sports-implied group viewed a product with sports-related elements (Figure 2A). Following this, we asked participants about their emotional responses to the packaging, such as: “This packaging makes me think of the vitality I feel during exercise.” Subsequently, we asked participants about their psychological empowerment using questions like: “For me, buying these products is extremely important” (1 = strongly disagree, 7 = strongly agree) (42) (Cronbach's α = 0.937; CMIN/DF = 1.995, GFI = 0.966, AGFI = 0.953, RMSEA = 0.041, CFI = 0.983, TLI = 0.980). We then used Pelletier et al. (43) exercise motivation scale to assess participants' intrinsic and extrinsic motivation, such as: “Because people I care about would be upset with me if I didn't” (extrinsic motivation) and “Because I have chosen this sport as a way to develop myself” (intrinsic motivation; 1 = strongly disagree, 7 = strongly agree; Cronbach's α = 0.92; CMIN/DF=3.811, GFI = 0.916, AGFI = 0.885, RMSEA = 0.069, CFI = 0.925, TLI = 0.908). Finally, participants answered questions regarding their purchase intention.

Referencing Yang et al. (40) research, we found that health consciousness is a critical factor influencing consumers' sustainable purchase intention. To further enhance the accuracy of the experiment, we also asked participants about their health consciousness with questions like: “I deeply care about my health” (1 = strongly disagree, 7 = strongly agree; Cronbach's α = 0.723). We also collected demographic information from the participants.

### 5.2 Experimental results

Manipulation check: we used emotional responses as the check variable and conducted an independent *t*-test. The results showed that participants in the sports-implied group perceived the product packaging as more emotionally engaging (*M*_traditionalpackaginggroup_ = 3.309, SD_traditionalpackaginggroup_ = 1.734; *M*_sports-*impliedgroup*_ = 4.263, SD_sports-*impliedgroup*_ = 1.627; *t* (1, 592) = −6.907, *P* < 0.001). Therefore, the manipulation in Experiment 2 was successful.

Primary effect test: we used product packaging type as the independent variable and purchase intention as the dependent variable, conducting a one-way ANOVA. The results indicated that participants' purchase intention for protein products with sports-implied packaging was significantly higher than for those with traditional packaging (*M*_traditionalpackaginggroup_ = 3.319, SD _traditionalpackaginggroup_ = 2.215; *M*_sports-*impliedgroup*_ = 4.902, SD _sports-*impliedgroup*_ = 1.358; *F*(1,592) = 110.145, *P* < 0.001). Thus, compared to traditional packaging, participants exhibited stronger purchase intention for protein products with sports-implied packaging, validating Hypothesis H1.

**Figure 3:**
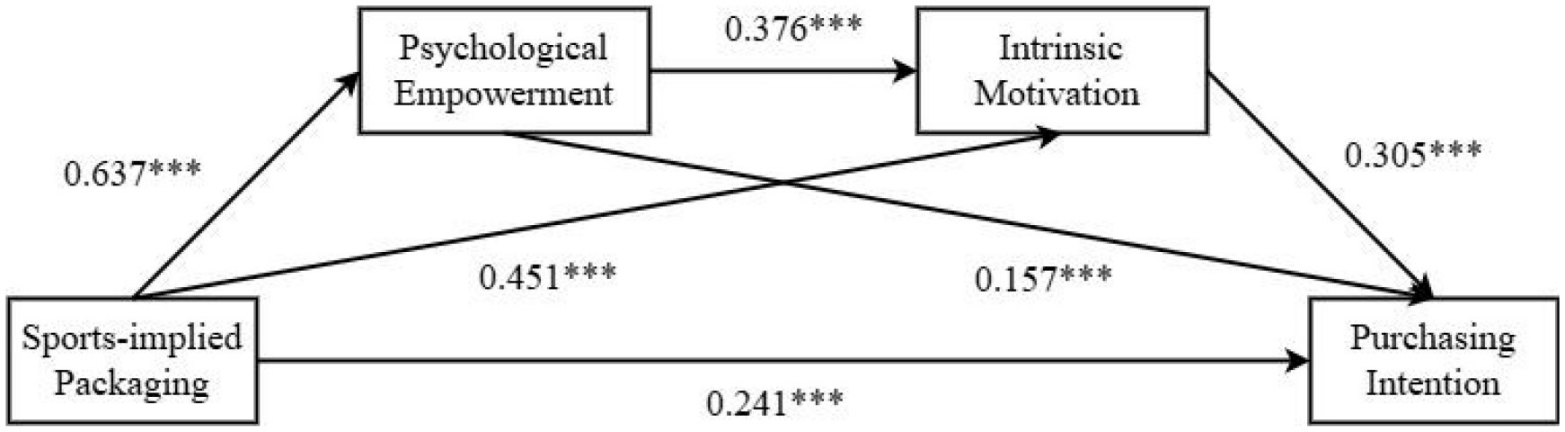
Path diagram of the chain mediation model with psychological empowerment and intrinsic motivation. ***P < 0.001.

Test of psychological empowerment and intrinsic motivation as chained mediators: we used product packaging type as the independent variable, purchase intention as the dependent variable, and intrinsic motivation and psychological empowerment as mediating variables. We applied Process Model 6 to test the chained mediating roles of intrinsic motivation and psychological empowerment (Bootstrap sample: 5,000) (44). Model 6 of the PROCESS Macro is to analyze the chain (sequence) mediation effect. It allows the examination of whether an independent variable influences the specific path of the dependent variable by affecting the first mediating variable, which in turn affects the second mediating variable. The core output is the confidence intervals of key indirect effects, in particular X → M1 → M2 → Y, calculated by the Bootstrap method to determine whether the path is significant. After controlling for health consciousness, the results indicated that the type of product packaging had a significant impact on psychological empowerment [β = 0.637, 95% CI = (0.481, 0.794), *P* < 0.001] and intrinsic motivation [β = 0.451, 95% CI = (0.301, 0.601), *P* < 0.001]; product packaging type also significantly influenced purchase intention [β = 0.241, 95% CI = (0.069, 0.412), *P* < 0.001]. Psychological empowerment significantly influenced intrinsic motivation [β = 0.376, 95% CI = (0.302, 0.449), *P* < 0.001) and purchase intention [β = 0.157, 95% CI = [0.069, 0.246], *P* < 0.001). Intrinsic motivation also significantly influenced purchase intention [β = 0.305, 95% CI = (0.215, 0.395), *P* < 0.001]. Overall, the path from product packaging type → psychological empowerment → intrinsic motivation → purchase intention was significant [β = 0.073, SE = 0.017, 95% CI = (0.044, 0.110)], as shown in Figure 3. Therefore, psychological empowerment and intrinsic motivation partially mediated the relationship between sports-implied packaging of protein products and Generation Z's purchase intention, validating Hypothesis H2a.

**Figure 4:**
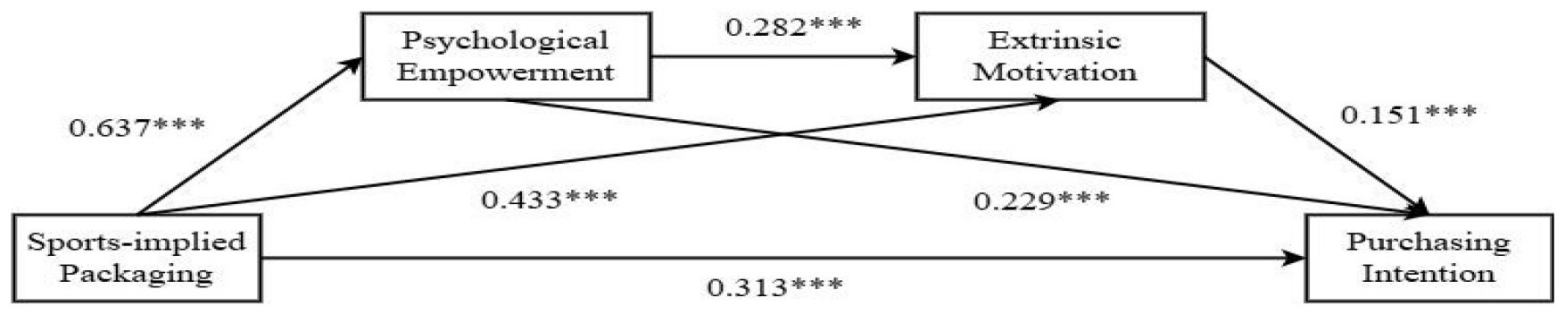
Path diagram of the chain mediation model with psychological empowerment and extrinsic motivation. ***P < 0.001.

Test of psychological empowerment and extrinsic motivation as chained mediators: we used extrinsic and psychological empowerment as mediating variables and applied Process Model 6 to test the chained mediating roles of extrinsic motivation and psychological empowerment (Bootstrap sample: 5,000) (44). After controlling for health consciousness, the results showed that product packaging type significantly influenced extrinsic motivation [β = 0.433, 95% CI = (0.267, 0.599), *P* < 0.001]. Psychological empowerment significantly influenced extrinsic motivation [β = 0.282, 95% CI = (0.200,0.364), *P* < 0.001], and extrinsic motivation significantly influenced purchase intention [β = 0.151, 95% CI = (0.068, 0.234), *P* < 0.001]. Overall, the path from product packaging type → psychological empowerment → extrinsic motivation → purchase intention was significant [β = 0.027, SE = 0.009, 95% CI = (0.011, 0.047)], as shown in Figure 4. Therefore, psychological empowerment and extrinsic motivation partially mediated the relationship between sports-implied packaging of protein products and Generation Z's purchase intention, validating Hypothesis H2b.

Comparison of indirect effects: we further compared the direct and indirect effects of the intrinsic and extrinsic motivation models, as shown in Table 4. The results indicated that, compared to psychological empowerment and extrinsic motivation [β = 0.027, SE = 0.009, 95% CI = (0.011, 0.047), Proportion of effect = 5%], the buffering effect of psychological empowerment and intrinsic motivation was more pronounced [β = 0.073, SE = 0.017, 95% CI = (0.044, 0.110), Proportion of effect = 13%], validating Hypothesis H3.

**Table 4:** Summary of indirect effects in the mediation models.

**Model**	**Effect type**	**β**	**SE**	**95% CI**	**Proportion of effect**
Extrinsic motivation model	Total effect	0.552	0.086	0.382, 0721	
	Direct effect	0.313	0.089	0.138, 0.488	57%
	Product packaging type - Psychological empowerment - Purchase intention	0.146	0.033	0.086, 0.215	26%
	Product packaging type - Extrinsic motivation - Purchase intention	0.065	0.023	0.026, 0.114	12%
	Product packaging type - Psychological empowerment - Extrinsic motivation - Purchase intention	0.027	0.009	0.011, 0.047	5%
Intrinsic motivation model	Total effect	0.552	0.086	0.382, 0.721	
	Direct effect	0.241	0.087	0.069, 0.412	44%
	Product packaging type - Psychological empowerment - Purchase intention	0.100	0.033	0.041, 0.168	18%
	Product packaging type - Intrinsic motivation - Purchase intention	0.138	0.030	0.084, 0.201	25%
	Product packaging type - Psychological empowerment - Intrinsic motivation - Purchase intention	0.073	0.017	0.044, 0.110	13%

### 5.3 Discussion

This study revealed how product packaging type influences Generation Z's purchase intention by conducting a chained mediation analysis, focusing on the mediating pathways of psychological empowerment and internal or external motivation. The results showed that sports-implied packaging enhances consumers' psychological empowerment, strengthening their intrinsic motivation and significantly promoting purchase intention. This partially supports Hypothesis H2a. Additionally, psychological empowerment indirectly influences purchase intention through extrinsic motivation, validating Hypothesis H2b. Notably, the comparison of indirect effects revealed that intrinsic motivation plays a stronger mediating role than extrinsic motivation, indicating that psychological empowerment is more likely to drive purchase intention by activating intrinsic rather than extrinsic motivators. This finding provides empirical evidence for Hypothesis H3. The results emphasize the critical role of psychological empowerment in marketing strategies and suggest that companies should enhance consumers' psychological empowerment through packaging design, particularly by activating their intrinsic motivation to improve conversion rates.

Despite the findings of Experiment 2, it did not further analyze the boundary conditions of the relationship between product packaging type and Generation Z's purchase intention. Therefore, in Experiment 3, we introduced disease threat as a moderating variable to examine the interaction between disease threat and product packaging type in influencing Generation Z's purchase intention.

## 6 Experiment 3: the moderating role of disease threat

### 6.1 Experimental design

Experiment 3 aimed to analyze the moderating role of disease threat in the relationship between sports-implied packaging of protein products and Generation Z's purchase intention. We designed a 2 (product packaging type: sports-implied vs. traditional packaging) × 2 (disease threat: high vs. low) factorial design and recruited 536 participants on a professional data collection platform. The demographic information for the participants is shown in Table 2. The 536 participants were randomly assigned to either the sports-implied or traditional packaging groups.

Experimental procedure: we asked both groups of participants to imagine the following scenario: In recent years, against the backdrop of frequent outbreaks of sudden public health events globally, people's tendency to stockpile dietary supplements has been increasing. At the time, they shop for protein nutrition products in a large commercial market. During their selection process, we showed the two groups different packaging designs for the same product. The traditional packaging group viewed a standard protein powder product, while the sports-implied group viewed a product with sports-related elements.

Simultaneously, we divided the sports-implied and traditional packaging groups into two subgroups: high and low disease threat groups. We presented the following stimulus material for the high disease threat group: “Emergency notice: monkeypox has reemerged as a global health threat. This is the second time monkeypox has been designated as a public health event of international concern since the World Health Organization declared it on July 23, 2022 (https://news.un.org/zh/story/2023/05/1117842).” For the low disease threat group, we presented: “Emergency notice: due to the increasing heat, water consumption has risen. From 9 a.m. to 12 p.m. tomorrow morning, water will be shut off in the city. Please prepare accordingly.” Finally, participants answered questions regarding their purchase intention.

### 6.2 Experimental results

Manipulation check: we used the perceived sense of dynamism and vitality as the check variable and conducted an independent *t*-test. The results showed that, compared to the traditional packaging group, participants in the sports-implied group perceived the product packaging as more dynamic and lively (*M*_traditionalpackaginggroup_= 3.847, SD _traditionalpackaginggroup_= 2.038; *M*_sports-*impliedgroup*_ = 4.649, SD _sports-*impliedgroup*_ = 1.721; *t* (1,534) = −4.924, *P* < 0.001). Therefore, the manipulation in Experiment 3 was successful.

Primary effect test: we used product packaging type as the independent variable and purchase intention as the dependent variable, conducting a one-way ANOVA. The results indicated that participants' purchase intention for protein products with sports-implied packaging was significantly higher than for those with traditional packaging (*M*_traditionalpackaginggroup_ = 4.179, SD _traditionalpackaginggroup_ = 1.923; *M*_sports-*impliedgroup*_ = 4.559, SD _sports-*impliedgroup*_ = 1.746; *F* (1,534) = 5.754, *P* = 0.017). Thus, compared to traditional packaging, participants exhibited stronger purchase intention for protein products with sports-implied packaging, validating Hypothesis H1.

**Figure 5:**
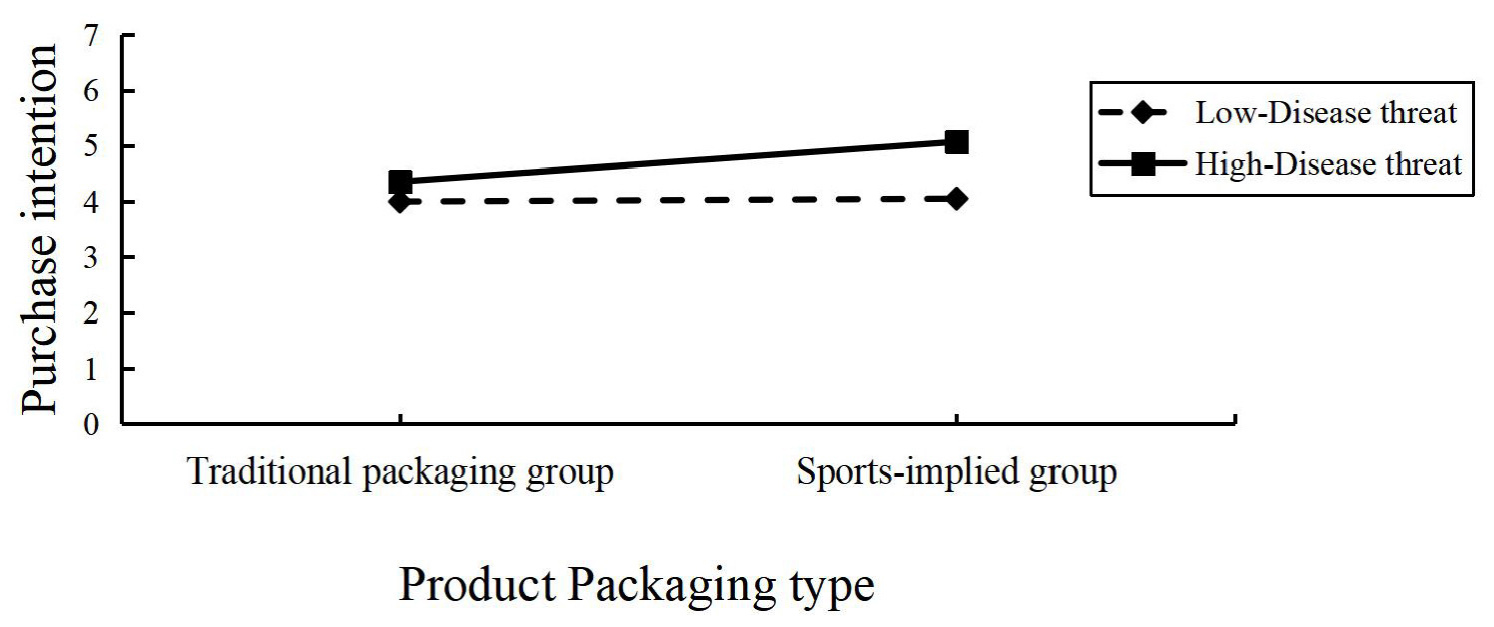
Interaction between product packaging type and disease threat on purchase intention.

Moderating effect test: we used disease threat as the moderating variable, product packaging type as the independent variable, and purchase intention as the dependent variable, conducting a two-way ANOVA. The results indicated that product packaging type significantly influenced purchase intention (*F*(1,534) = 5.990, *P* = 0.015); disease threat also significantly influenced purchase intention (*F*(1,534) = 19.493, *P* < 0.001); and the interaction between disease threat and product packaging type significantly influenced purchase intention (*F*(1,534) = 4.459, *P* = 0.035), as shown in Figure 5. Therefore, disease threat significantly moderated the relationship between product packaging type and purchase intention.

### 6.3 Discussion

The moderating effect test demonstrated that disease threat significantly influenced the relationship between packaging type and purchase intention. This finding highlights the critical role of health-related motivations in consumer decision-making, particularly in the context of sports nutrition products. When a disease threat is salient, the appeal of sports-implied packaging is amplified, as consumers are more inclined to prioritize health-enhancing products. This result underscores the importance of leveraging visual and emotional cues in packaging design to align with consumer health consciousness and risk-averse tendencies. These findings underscore the strategic value of sports-implied packaging as a marketing tool. By integrating dynamic, health-related visual elements, brands can create packaging designs that not only stand out in a competitive market but also resonate deeply with the psychological and behavioral drivers of Generation Z consumers.

## 7 General discussion

This study provides a new perspective on how sports-implied packaging (vs. traditional packaging) influences consumer purchase intention in sports nutrition, specifically for protein products. The results confirm that protein products utilizing sports-implied packaging are more effective in stimulating purchase intention (H1), an effect partially mediated by psychological empowerment and exercise motivation (H2 and H3), and moderated by disease threat (H4). These findings contribute to both advertising theory and marketing practices, particularly in the development of innovative packaging strategies for nutritional products.

Our empirical data from three experiments reveal nuanced pathways in consumer motivation. Notably, the chained mediation analysis in Experiment 2 highlights that psychological empowerment acts as an initial catalyst, enhancing exercise motivation, which in turn drives purchase intention. However, the paths diverge in strength: the intrinsic motivation route proves more robust, accounting for 13% of the total effect, compared to only 5% for extrinsic motivation. This suggests that when sports-implied packaging evokes internal drives—such as personal growth and enjoyment in fitness—Generation Z consumers experience a deeper, more enduring pull toward purchase, as opposed to externally driven factors like social approval. Linking to self-determination theory, this disparity arises because intrinsic motivation directly satisfies core needs for autonomy and competence, promoting sustained behavioral commitment without the diminishing returns associated with extrinsic pressures (28). In practical terms, this implies that packaging designs fostering self-fulfillment (e.g., imagery of personal achievement) yield stronger consumer responses than those implying external validation, offering a pathway for long-term brand engagement in health markets.

Furthermore, the moderating effect of disease threat in Experiment 3 amplifies these dynamics under high-threat conditions, where purchase intention surges for sports-implied packaging, underscoring how external stressors heighten the appeal of empowerment-driven cues. Overall, these descriptive insights move beyond mere hypothesis validation to illuminate actionable mechanisms for sensory-driven marketing in volatile health contexts.

### 7.1 Theoretical implications

This study is the first to introduce the relationship between sports-implied packaging and consumer purchase intention into the field of sports nutrition psychology, demonstrating through empirical research that protein powder products with sports-implied elements are more effective in triggering purchase intention among Generation Z consumers compared to traditional packaging. These findings hold significant theoretical importance for consumer behavior research.

First, this research identifies sports imagery as an important visual cue in sensory marketing, particularly for health products. Traditional visual marketing studies have primarily focused on how packaging elements such as color, typography, and imagery influence consumer preferences (45, 46). In contrast, this study reveals sports imagery—encompassing dynamic athletic scenes, motivational symbols, and vitality-evoking designs—as a distinct cue that activates embodied health associations and priming effects, thereby enhancing product appeal in the sports nutrition domain (47). By conveying signals of vitality, performance, and self-improvement, sports imagery aligns more effectively with Generation Z's health-oriented values and lifestyle aspirations, differentiating it from generic or informational visuals in traditional packaging. This precise identification enriches sensory marketing theory by specifying how targeted visual cues can evoke automatic cognitive and emotional responses, such as reduced decision latency and heightened self-efficacy, in health-focused consumption contexts (48).

Through comparative analysis, sports-implied packaging demonstrates a more substantial advantage over traditional packaging in triggering purchase intention, indicating that consumer purchasing decisions are influenced not only by the functional attributes of a product but also by the emotional and lifestyle values conveyed through packaging. This finding further validates the core tenets of product experience theory, which posits that purchasing behavior results from a combination of sensory, perceptual, and emotional experiences (49). By introducing the concept of sports-implied packaging, this study reveals the mediating role of visual experience in consumer purchasing decisions, providing new empirical support for product experience theory.

This study reveals the mediating role of psychological empowerment in consumer purchasing behavior, expanding the application of psychological empowerment theory. Traditional psychological research has primarily viewed psychological empowerment as a psychological state that explains self-efficacy in work, education, or health behaviors (50). However, this study introduces psychological empowerment into consumer behavior, demonstrating its significant influence on purchasing decisions through its enhancement of intrinsic motivation and behavioral intentions. This discovery not only enriches the theoretical understanding of psychological empowerment but also opens new avenues for applying psychological empowerment theory to consumer behavior research.

Moreover, this research highlights the critical role of exercise motivation as a mediator between psychological empowerment and purchase intention, making an important contribution to the refinement of exercise psychology theories. Self-determination theory emphasizes that intrinsic motivation is a core driver of human behavior (28). This study demonstrates that sports-implied packaging enhances consumers' psychological empowerment, strengthening their exercise motivation and ultimately increasing purchase intention. This finding further validates the applicability of self-determination theory in the context of consumer behavior and elucidates the transmission mechanism by which exercise motivation shapes purchasing behavior.

The study's finding that the indirect effect of psychological empowerment and intrinsic motivation is more pronounced than extrinsic motivation holds significant theoretical value for developing exercise psychology, consumer psychology, and motivation theory. Specifically, our results indicate that the chained mediation path via intrinsic motivation is stronger than via extrinsic motivation. This disparity aligns with self-determination theory, which posits that intrinsic motivation—driven by inherent interest, autonomy, and competence satisfaction—is a more powerful and sustainable driver of behavior compared to extrinsic motivation, which relies on external rewards or pressures (28, 29). In the context of health product consumption, intrinsic motivation directly fulfills psychological needs, fostering long-term engagement and reducing reliance on transient incentives (30). For Generation Z, this manifests as heightened purchase intention when packaging evokes self-directed fitness goals, rather than social obligations. The empirical validation of this theory in the context of consumer behavior underscores its relevance to marketing strategies, emphasizing the need for designs that prioritize internal fulfillment over external prompts.

Finally, this study's revelation of the moderating role of disease threat in consumer purchasing behavior expands the application of the health belief model in consumer psychology (51). Traditionally, the HBM has been used to explain the motivations and decisions behind health-related behaviors. However, this study applies the model to consumer purchasing behavior, demonstrating that disease threat influences purchase intention by intensifying consumers' demand for health products. Generation Z consumers, characterized by heightened health awareness and risk aversion, are exceptionally responsive to health and safety information. This study's finding that disease threat significantly moderates Generation Z's purchase intention offers a new theoretical perspective for understanding their health-related consumption behaviors.

### 7.2 Practical implications

This study's finding that sports-implied packaging generates stronger purchase intention among Generation Z consumers than traditional packaging provides practical guidance for packaging design and marketing strategies. Generation Z, as the primary consumer group in the digital age, is deeply influenced by individualization, interactivity, and visual experiences. Sports-implied packaging, through the integration of sports-related visual elements, motivational language, and health-related symbols, aligns more effectively with the values and lifestyle of Generation Z consumers. Specifically, companies can incorporate images of sports scenes, color schemes associated with sports, or motivational phrases into their packaging designs to trigger consumers' health consciousness and purchasing desire. Such packaging not only enhances the product's visual appeal but also strengthens consumers' perceived functionality through psychological suggestion, thereby increasing purchase intention. Thus, this study provides practical evidence for companies to incorporate sports-implied elements into protein product packaging, helping them stand out in competitive markets.

The chained mediating roles of psychological empowerment and exercise motivation offer crucial insights for companies on activating consumers' intrinsic motivation through packaging design and marketing strategies. Psychological empowerment reflects consumers' sense of self-efficacy and control during the process of purchasing and using a product. At the same time, exercise motivation represents consumers' internal drive to engage in physical activities to achieve health goals. Companies can further enhance consumers' psychological empowerment by incorporating phrases such as “designed specifically for your fitness goals” or “helping you achieve a healthy lifestyle” into packaging designs, or by providing personalized health guidance through interactive digital features. Additionally, companies can stimulate consumers' exercise motivation through sports-implied packaging by showcasing sports-related imagery or success stories, conveying the product's support for consumers' health objectives. By leveraging this chained mediating mechanism, companies can more precisely align with Generation Z's intrinsic needs, enhancing purchase intention and brand loyalty.

The discovery that psychological empowerment and intrinsic motivation have a more pronounced indirect effect than external motivation holds important implications for companies focusing on intrinsic motivation in consumer behavior guidance and marketing strategies. Generation Z consumers are characterized by strong individualization and self-expression needs, making them more likely to choose products that help them achieve personal values and health goals. Intrinsic motivation is more likely to sustain long-term interest and purchasing behavior than external motivation. Therefore, companies should prioritize consumers' intrinsic needs in packaging design and marketing, rather than relying excessively on external incentives. For instance, sports-implied packaging can convey values such as health, vitality, and self-realization, helping consumers associate the product with their personal goals and enhancing purchase intention. Furthermore, companies can reinforce consumers' intrinsic motivation through brand storytelling or corporate social responsibility initiatives, boosting purchase intention and strengthening consumers' identification with and loyalty to the brand.

The finding that disease threat significantly moderates the relationship between sports-implied packaging and Generation Z's purchase intention offers important insights for companies regarding health-related advertising and crisis communication strategies. Generation Z consumers are highly attentive to health issues and seek to prevent disease through their behaviors. Companies can emphasize the health attributes of their protein products in sports-implied packaging, such as highlighting the product's benefits for immune enhancement, muscle repair, or cardiovascular health. This allows consumers to view the product as a proactive solution to potential health threats. Furthermore, companies can emphasize the health benefits of their products in marketing campaigns, such as through health claims on packaging or in advertisements. Such health-oriented packaging and advertising strategies not only meet Generation Z's health needs but also enhance a company's competitive edge in a crowded market.

### 7.3 Research limitations and future research development directions

This study, while providing valuable insights into the effects of sports-implied packaging on Generation Z's purchase intentions, is subject to several significant limitations that constrain its generalizability and validity. This study mainly surveyed Generation Z consumers. The sample range was relatively small and failed to cover consumers of other age groups or cultural backgrounds. The values and behavioral patterns of Generation Z consumers may differ significantly from those of other groups, which limits the universality of the research results to a certain extent. Furthermore, this study's sample size and geographical scope may not be sufficient to fully represent the consumer groups in a global or multicultural environment.

More critically, the use of online scenario-based experiments with purchased panels poses substantial challenges to external validity. Participants were recruited from professional data collection platforms and compensated with a small remuneration (3 CNY), which may have led to low task engagement or interest in the stimuli. It remains unclear whether respondents were genuinely motivated to imagine purchasing protein powder products, as opposed to hastily completing the survey for payment. This artificial setting deviates from real-world shopping environments, where contextual factors such as in-store displays, social influences, or immediate product interactions could alter decision-making processes (52). Additionally, reliance on self-reported measures introduces potential common method bias, inflating relationships between variables (53). Although we employed attention checks and excluded invalid responses, these issues could compromise the ecological validity of our findings.

Another severe limitation is the focus on a single product category—protein powder—which restricts generalization to other nutritional supplements or food products. Protein powder is inherently linked to fitness goals, potentially amplifying the effects of sports-implied cues; similar packaging strategies might not yield comparable results for less sport-related items, such as vitamins or energy drinks (54). This narrow scope limits the broader applicability of our theoretical model to diverse consumer goods.

Future research can expand the sample range to include consumers of different ages, genders, cultural backgrounds and geographical regions to verify the cross-cultural applicability of the effect of sports suggestion packaging. In addition, packaging design strategies for different consumer groups can also be further explored to optimize the acceptance of products in the global market.

This study adopted an experimental design to verify the influence of sports-implied packaging on consumers' purchase intention. However, the experimental conditions may not be able to fully simulate the real purchasing environment. Consumers' decisions in the laboratory may differ from their behaviors in real shopping scenarios. Furthermore, this study mainly collected data through questionnaire surveys. The questionnaire design may have subjective biases and cannot fully capture the true psychological state and behavioral intentions of consumers. Future research can combine empirical data (such as purchase records, eye-tracking techniques or neuroscience methods) with experimental designs to further verify the impact of motion-suggestive packaging on consumer behavior. Meanwhile, introduce more ecologically effective research environments (such as virtual reality shopping scenarios) to enhance the external effects of the research results. To address the limitations in external validity, future studies should incorporate real purchase data or behavioral measures, such as analyzing click-through rates on online advertisements featuring sports-implied packaging or tracking actual sales data from e-commerce platforms. This would provide objective evidence of behavioral outcomes beyond self-reported intentions.

Although this study proposed the chain mediating effect of psychological empowerment and exercise motivation, other potential mediating or moderating variables might have been overlooked in the model, such as consumers' personality traits (such as pioneering new styles), social influence or cultural values, etc. These variables may play an important role in the effect of motion suggestion packaging. Furthermore, the study only focused on disease threat as a moderating variable, while other moderating factors (such as price, brand loyalty, or product functionality) might also have an impact on the effect of sports suggestion packaging. Future research can further expand the theoretical model, introduce more potential variables, and construct a more complex and comprehensive theoretical framework to explain the diversity of consumer purchasing behavior. For instance, it is possible to explore how price sensitivity or brand trust can regulate the effect of campaign-suggestion packaging, thereby providing enterprises with a more comprehensive marketing strategy.

Building on embodied cognition theory, researchers could investigate other embodied cues in packaging, such as the weight of the container or tactile textures (e.g., grippy surfaces evoking sports equipment), to determine their additive or interactive effects on exercise motivation and purchase intentions (55). Finally, given the Chinese-centric sample, exploring cultural differences in responses to sports-implied cues—such as individualistic vs. collectivistic orientations (56)—would enhance the model's cross-cultural robustness and inform global marketing strategies.

## 8 Conclusion

This study recruited 1,456 participants and conducted three experiments to verify that the sports-implied packaging of protein powder products was more effective in stimulating Generation Z's purchasing intention than traditional packaging. During this process, psychological empowerment and exercise motivation can strengthen this set of relationships, and the threat of disease effectively moderates the relationship between exercise suggestion packaging and purchase intention.

## Data Availability

The original contributions presented in the study are included in the article/Supplementary material, further inquiries can be directed to the corresponding author.
