# Supplementary material for: How sports-implied packaging of protein powder products enhances the purchase intention of Generation Z: evidence from multiple experiments
Source: Front Nutr. 2025 Nov 14;12:1645614. doi: 10.3389/fnut.2025.1645614 (PMC12661654; doi:10.3389/fnut.2025.1645614)
Supplement: Supplementary file 1 [file Supplementary_file_1.pdf]

Appendix

The measurement title of the variables used in this study.

Variables	Items
Perceived sense of dynamism	When you see the above product, do you agree that its packaging is dynamic and upbeat?
Emotional responses	This packaging makes me think of the vitality I feel during exercise.
Psychological empowerment	For me, purchasing these products is very important.
	I am confident of my ability to consume these products
	I have the needed competence to use these products.
	I feel pleased when I contribute to the protection of the environment by buying these products.
	I feel pleased when i consume these products because they are close to nature.
	I feel pleased when I consume like our ancestors did
	I like bio products
	Consumption of these products allows me to live longer.
	I feel in good health when I consume these products
	I consume these products to reduce risk of getting ill
	I consume these products for their unique taste
	I consume these products to avoid all that is chemical.
	The impact of consuming these products on my entourage is important.
	By consuming these products, i feel I control the events in my entourage.
Extrinsic motivation	Because people I care about would be upset with me if I didn't
	Because I think others would disapprove of me if I did not
	Because people around me reward me when I do.
	I used to have good reasons for doing sports, but now I am asking myself if I should continue.
	So that others will praise me for what I do.
	It is not clear to me anymore; I don't really think my place is in sport.
Intrinsic motivation	Because it gives me pleasure to learn more about my sport.
	Because I find it enjoyable to discover new performance strategies.
	Because it is very interesting to learn how I can improve.
	Because practicing sports reflects the essence of whom I am.
	Because participating in sport is an integral part of my life
	Because through sport, I am living in line with my deepest principles.
	Because I have chosen this sport as a way to develop myself.
	Because I found it is a good way to develop aspects of myself that I value
	Because it is one of the best ways I have chosen to develop other aspects of myself.
	Because I would feel bad about myself if I did not take the time to do it.

	Because I feel better about myself when I do.
	Because I would not feel worthwhile if I did not.
Health consciousness	I care deeply about your health.
	I regularly reflect on my health.
	I are usually very mindful of how I feel inside about my health.
Purchase intention	When you see the above product, do you agree that you would consider purchasing this product?
